# Nesfatin-1 promotes brown adipocyte phenotype

**DOI:** 10.1038/srep34747

**Published:** 2016-11-08

**Authors:** Yuexin Wang, Ziru Li, Xinyu Zhang, Xinxin Xiang, Yin Li, Michael W. Mulholland, Weizhen Zhang

**Affiliations:** 1 Department of Physiology and Pathophysiology, Peking University Health Science Center and Key Laboratory of Molecular Cardiovascular Science, Ministry of Education, Beijing 100191, China; 2 Department of Surgery, University of Michigan Medical Center, Ann Arbor, MI 48109, USA

## Abstract

Nesfatin-1, an 82 amino acid gastric peptide, is involved in regulation of food uptake and in multiple metabolic activities. Whether nesfatin-1 modulates the differentiation and lipid metabolism of brown adipocytes remains unknown. In the present study, we found that nesfatin-1 mRNA and protein were detectable in isolated brown adipocytes and gradually decreased during differentiation (95% CI 0.6057 to 1.034, p = 0.0001). The decrease in nesfatin-1 was associated with a significant reduction in p-S6. Exposure to nesfatin-1 promoted differentiation of brown adipocytes as revealed by a significant increase in UCP1 mRNA (p = 0.03) and lipolysis-related ATGL mRNA (p = 0.04). Nesfatin-1 attenuated phosphorylation of S6K and S6 during brown adipocyte differentiation. Activation of mTOR by leucine or deletion of TSC1 decreased expression of brown adipocyte-related genes UCP1, UCP3, PGC1α and PRDM16, as well as COX8B and ATP5B. Both leucine and TSC1 deletion blocked nesfatin-1-induced up-regulation of UCP1, PGC1α, COX8B and ATP5B in differentiated brown adipocytes. In conclusion, nesfatin-1 promotes the differentiation of brown adipocytes likely through the mTOR dependent mechanism.

Brown adipose tissue is involved in thermogenesis, in contrast to white adipose tissue which serves as an organ to store excess energy. Unlike white adipocytes which are characterized by large lipid droplets, brown adipocytes are smaller in size and contain multilocular droplets of lipid and large mitochondria with cristae^1^. The characteristic mitochondria within brown adipocytes contain uncoupling protein 1(UCP1) which functions in thermogenesis instead of energy storage^2^. Brown adipose tissue also regulates blood lipid homeostasis by taking up triglyceride^3^,^4^. Additionally, brown adipose tissue regulates circulating glucose to ameliorate glucose tolerance under cold exposure^5^. Brown adipose tissue transplantation improves glucose tolerance and insulin sensitivity^6^.

Nesfatin-1 is an 82 amino acid peptide originally localized to hypothalamic nuclei which inhibits nocturnal food uptake. Nesfatin-1 is also expressed in peripheral tissues such as gut, pancreas islet, and testis^7^,^8^. In addition to anorexic effect, nesfaitn-1 has been reported to regulate lipid metabolism, insulin secretion and glucose homeostasis, stress responses, and reproduction^9^. These actions are proposed to be mediated through a central mechanism, with the stomach as the main source of circulating nesfatin-1. While the effect of nesfatin-1 on brown adipose tissue is unknown, cold exposure activates nesfatin-1-positive neurons within brain regions critical for the regulation of thermogenesis, suggesting a potential relationship between circulating nesfatin-1 and function of brown adipose tissue^10^.

We report the production of nesfatin-1 by brown adipocytes. Levels of nesfatin-1 decrease during differentiation of brown adipocytes. Further, nesfatin-1 promotes the brown adipocyte phenotype, in part by inhibiting mTOR signaling.

## Results

### Differentiation of primary brown adipocytes

**Figure 1:**
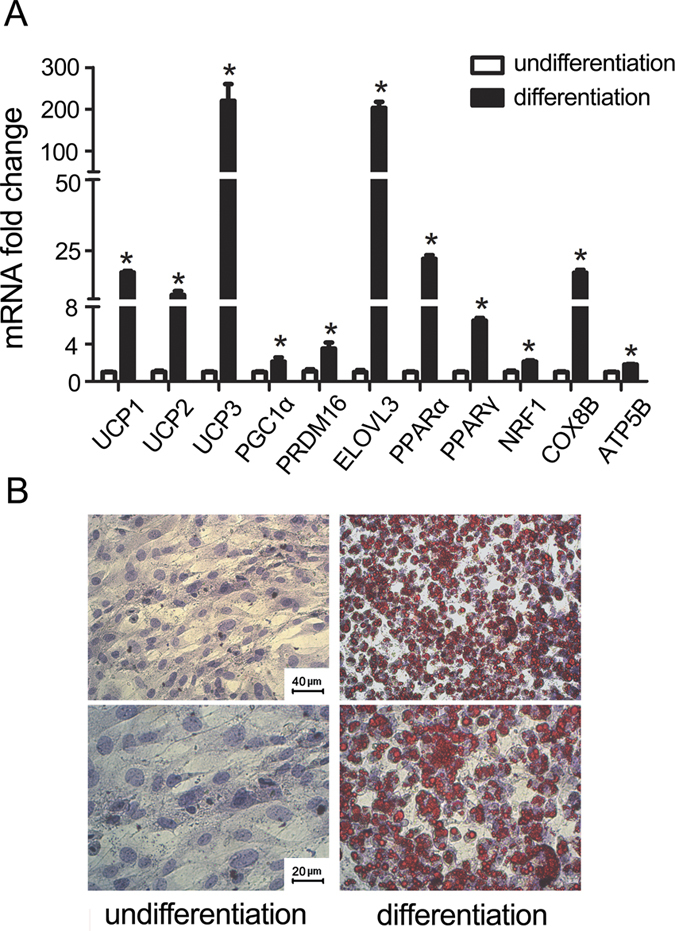
Effective differentiation of brown adipocytes. (**A**) Total RNAs were extracted from primary brown adipocytes after four days of differentiation, Real-time PCR was performed to evaluate the expression of brown adipocyte marker genes. (**B**) Oil red O staining was performed to demonstrate the lipid droplets.

To demonstrate the effective differentiation of brown adipocytes in our experiment, brown adipocyte gene markers and oil red O lipid droplets were examined after adding differentiation medium into the culture brown preadipocytes for four days. As shown in Fig. 1A, brown adipocyte gene markers mRNA were significantly increased after induced differentiation (p < 0.05). Multilocular lipid droplets were detected by oil red O staining (Fig. 1B), indicating the effective differentiation of brown adipocytes.

### Decrease of nesfatin-1 during differentiation of brown adipocytes

**Figure 2:**
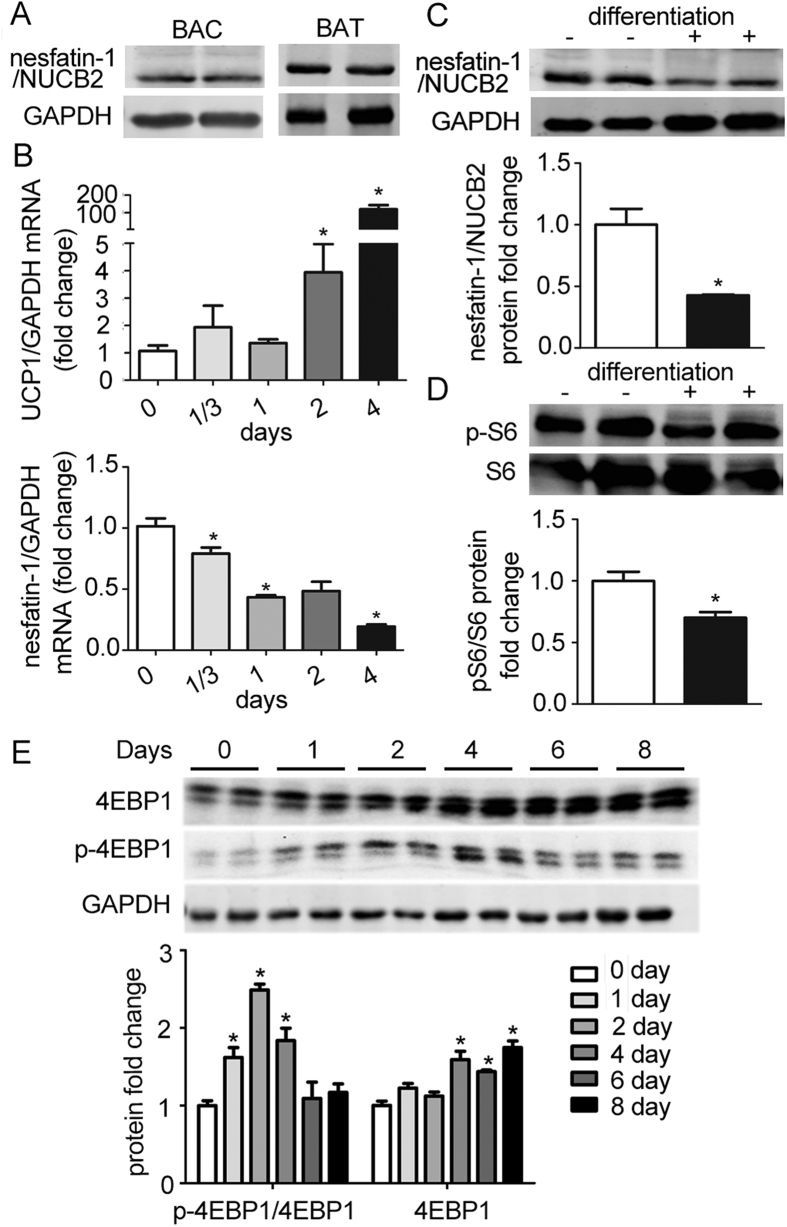
Decrease of nesfatin-1 and mTOR signaling activity during the differentiation of brown adipocytes. (**A**) Proteins were extracted from primary brown adipocytes (BAC) and interscapular brown adipose tissue (BAT). Western blotting of NUCB2/nesfaitn-1 was performed with GAPDH used as a loading control. (**B**) Total RNAs were extracted from primary brown adipocytes at different time points of differentiation. Real-time PCR were performed to evaluate the expression of UCP1 and nesfatin-1. GAPDH was used as internal control. (**C**,**D**) Representative results of western blot for NUCB2/nesfaitn-1 and phosphorylated S6. GAPDH was used as the loading control for the western blot. Relative protein signal intensity was quantified, and expressed as mean ± SEM. *P < 0.05 (n = 3–4). (**E**) Representative results of western blot for p-4EBP1 during differentiation process. GAPDH was used as the loading control. Relative protein signal intensity was quantified, and expressed as mean ± SEM. *denotes P < 0.05 relative to undifferentiated control (n = 3–4).

As shown in Fig. 2A, NUCB2/nesfatin-1 protein was detected in both cultured brown adipocytes and interscapular brown adipose tissue from C57/BL6J mice. To investigate whether the expression of nesfatin-1 changes during the differentiation of brown adipocytes, differentiation of primary brown adipocytes was induced as described in the methods section, and levels of nesfatin-1 mRNA were detected at 8 h, 1, 2, and 4 days. During the differentiation of brown adipocytes, as measured the up-regulation of UCP1 mRNA, levels of nesfatin-1 mRNA were significantly decreased (Fig. 2B). Consistent with change in mRNA levels, nesfatin-1 protein decreased (p < 0.05) after induction of differentiation of brown adipocytes (Fig. 2C).

To investigate whether the mTOR signaling pathway was involved in the differentiation of brown adipocytes, we examined phosphorylation of S6 and 4EBP-1. As shown in Fig. 2D, levels of S6 phosphorylation were significantly down-regulated on day 4 after induction of differentiation of primary brown adipocytes. During differentiation of brown adipocytes, phosphorylation of 4EBP-1 demonstrated a temporal alteration. Its levels increased significantly from day 1 to day 4, followed by subsequent down-regulation on day 6 and day 8 (Fig. 2E).

### Nesfatin-1 promotes differentiation of brown adipocytes and lipolysis

**Figure 3:**
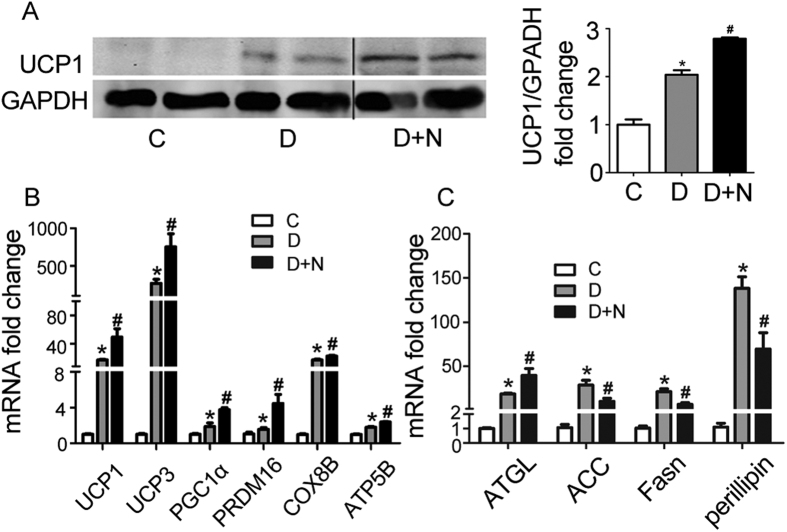
Effects of nesfatin-1 on the differentiation of brown adipocytes and their lipid metabolism. Primary brown adipocytes were isolated from neonatal C57BL/6J mice and cultured for differentiation. (**A**) Representative results of western blot for UCP1. GAPDH was used as the loading control. Relative protein signal intensity was quantified, and expressed as mean ± SEM. (**B**,**C**) Real-time PCR were performed to evaluate the expression of brown adipocyte genes related to differentiation and lipid metabolism. Levels of mRNA expression were normalized to GAPDH, and expressed as mean ± SEM. *P < 0.05 vs. control without differentiation (n = 3–4); ^#^P < 0.05 vs. differentiation without nesaftin-1 (n = 3–4). Each experiment was repeated at least 3 times. C: control; D: differentiation; N: nesfatin-1.

To investigate whether nesfatin-1 affects the differentiation of brown adipocytes, nesfatin-1 at a concentration of 10^−8^ mol/L was added to the differentiation medium of primary brown adipocytes. As shown in Fig. 3A, UCP1 protein, a brown adipocyte marker, increased 42 ± 9% upon exposure to nesfatin-1 relative to brown adipocytes without nesfatin-1 stimulation (p = 0.02). The mRNA levels of brown adipocyte markers: UCP1 (p = 0.03), UCP3 (p = 0.04), PGC1α (p = 0.01), and PRDM16 (p = 0.03), COX8B (p = 0.018) and ATP5B (p = 0.011) dramatically increased compared to brown adipocytes without nesfatin-1 exposure (Fig. 3B). These results suggest that nesfatin-1 promotes the differentiation of primary brown adipocytes. To further investigate whether nesfatin-1 affects lipid metabolism in primary brown adipocytes, we examined the expression levels of genes relevant to lipid metabolism. As shown in Fig. 3C, ATGL, a gene related to lipolysis, increased significantly (p = 0.04), while genes related to lipogenesis such as ACC (p = 0.01) and Fasn (p = 0.04) demonstrated significant decreases. In addition, perilipin (p = 0.03) mRNA was down-regulated by nesfatin-1.

### Nesfatin-1 attenuates mTOR signaling

**Figure 4:**
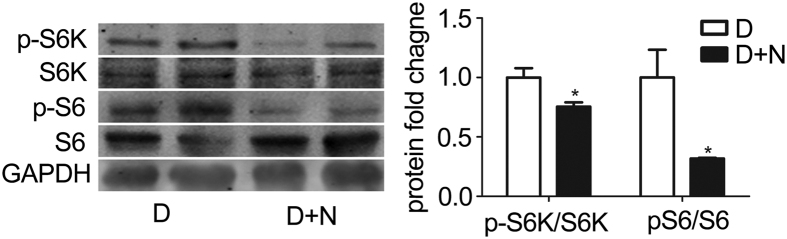
Nesfatin-1 attenuates mTOR signaling pathway. Protein was extracted from primary brown adipocyte as described. Western blotting was performed. Representative results of western blotting for p-S6K1, S6K, p-S6, and S6 are shown. GAPDH was used as loading control. Relative protein signal intensity was quantified, and expressed as mean ± SEM. *P < 0.05 vs. differentiation. Each experiment was repeated at least 3 times. D: differentiation; N: nesfatin-1.

mTOR signaling pathway has been demonstrated to mediate the effects of nesfatin-1 on food intake in medullar neurons. In addition, mTOR activity is inhibited during differentiation of brown adipocytes (Fig. 2D). We thus investigated the effects of nesfatin-1 on mTOR signaling in cultured brown adipocytes by examining the phosphorylation of S6K and S6, downstream targets of mTOR. As shown in Fig. 4, nesfatin-1 significantly attenuated phosphorylation of S6K and S6 in brown adipocytes.

### mTOR signaling mediates differentiation of brown adipocytes

**Figure 5:**
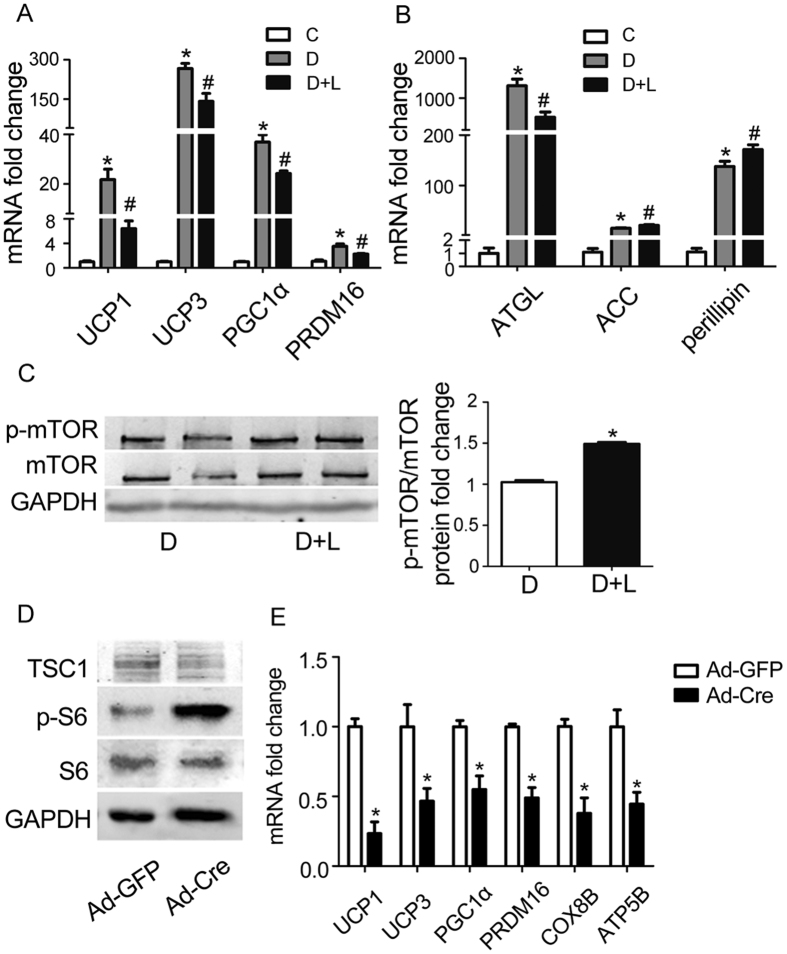
Activation of mTOR signaling inhibits the differentiation of primary brown adipocytes. (**A**,**C**) Cultured brown adipocytes were treated with leucine (5 mM). RNAs and protein were extracted as described in the method section. Western blot was performed to evaluate the alternation phosphorylated mTOR. GAPDH was used as loading control. Relative protein signal intensity was quantified, and expressed as mean ± SEM. Real-time PCR was performed to evaluate the expression of genes related to brown adipocyte differentiation and lipid metabolism. Levels of mRNA expression were normalized to GAPDH, and expressed as mean ± SEM. *P < 0.05 vs. control without differentiation (n = 3–4); ^#^P < 0.05 compared to differentiation without leucine (n = 3–4). Each experiment was repeated at least 3 times. C: control; D: differentiation; N: nesfatin-1; L: leucine. A Genes related to brown adipocyte differentiation. Representative results of western blotting for p-mTOR, mTOR. (**B**) Genes related to lipid metabolism. (**C**) Representative results of western blotting for p-mTOR, mTOR. (**D**,**E**) *Tsc1*^*loxp/loxp*^brown adipocytes were treated with Cre adenoviruses for 48 hours, while control used GFP adenoviruses. RNAs and protein were extracted as described in the method section. Western blot was performed to evaluate the alternation of Tsc1 and phosphorylated S6. GAPDH was used as loading control. Real-time PCR was performed to evaluate the expression of genes related to brown adipocyte differentiation. Levels of mRNA expression were normalized to GAPDH, and expressed as mean ± SEM. *P < 0.05 (n = 4). Each experiment was repeated at least 3 times.

To determine the role of mTOR signaling in brown adipocyte differentiation, we treated primary brown adipocytes with leucine (5 mM), a branch-chain amino acid that has been documented to activate mTOR signaling, and examined mRNA levels of brown adipocyte marker genes. As shown in Fig. 5A, treatment of cultured brown adipocytes significantly increased the phosphorylation of mTOR, indicating the activation of mTOR signaling in these cells. The leucine-induced activation of mTOR signaling was associated with a significant decrement in mRNA levels of brown adipocyte markers such as UCP1 (p = 0.01), UCP3 (p = 0.02), PGC1α (p = 0.01) and PRDM16 (p = 0.04) (Fig. 5B). mRNA levels of ATGL was significantly down-regulated (p = 0.01), while mRNA levels of ACC (p = 0.03) and perilipin (p = 0.04) were up-regulated in response to leucine exposure (Fig. 5C).

To further demonstrate the effects of mTOR on the differentiation of brown adipocytes, brown preadipocytes were isolated from *Tsc1*^loxp/loxp^ transgenic mice and treated with Cre adenovirus to delete the tuberous sclerosis complex 1 (*Tsc1*) gene, the upstream inhibitor of mTOR signaling. As shown in Fig. 5D, deletion of *Tsc1* significantly increased the phosphorylation of S6 relative to the control Ad-GFP group, indicating the activation of mTOR signaling pathway in these cells. Activation of mTOR caused a significant decrease in brown adipocyte marker gene, such as UCP1 (p = 0.003), UCP3 (p = 0.043), PGC1α (p = 0.006), PRDM16 (p = 0.003), COX8B (p = 0.0009), ATP5B (p = 0.006) (Fig. 5E). These results indicate that up-regulation of mTOR signaling attenuates the differentiation of primary brown adipocytes

### mTOR dependent effects of nesfaitn-1

**Figure 6:**
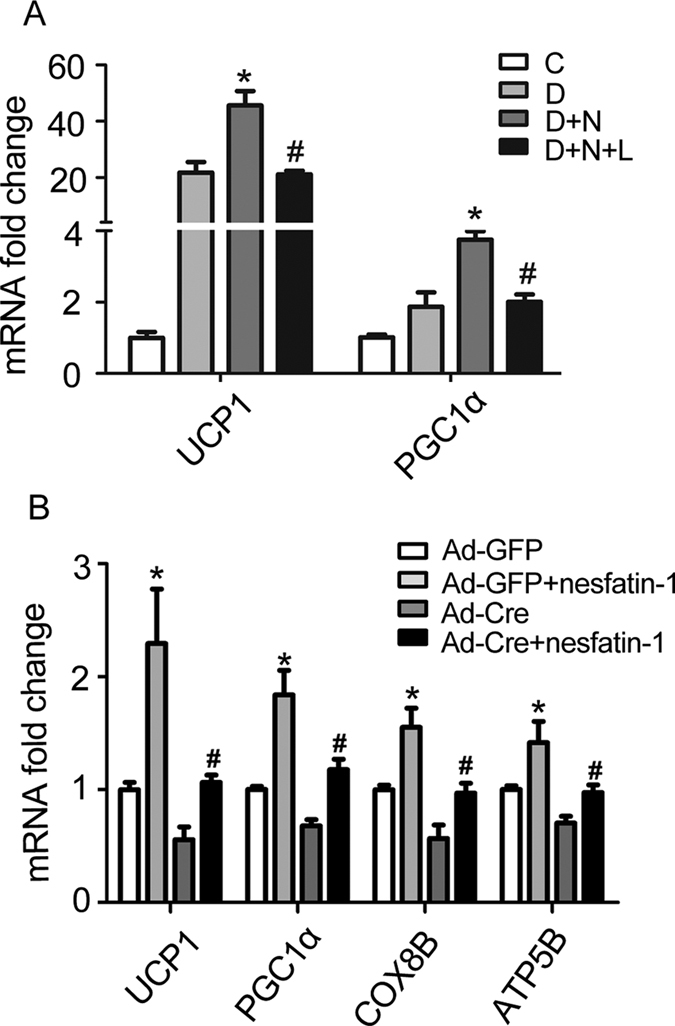
mTOR dependent effects of nesfaitn-1. (**A**) Effects of leucine. Nesfatin-1 alone or together with leucine was administrated to cultured brown adipocyte. Real-time PCR was performed to evaluate the expression of brown adipocyte genes related to differentiation. The mRNA levels of UCP1 and PGC1α were presented. Levels of mRNA expression were normalized to internal control GAPDH, expressed as mean ± SEM. *P < 0.05 vs. differentiation (n = 3–4); ^#^P < 0.05 vs. differentiation with nesfatin-1 (n = 3–4). Each experiment was repeated at least 3 times. (**B**) Effects of *Tsc1* deletion. Cre or GFP adenoviruses were administrated to cultured brown adipocytes for 48 hours. Real-time PCR was performed to evaluate the expression of brown adipocyte genes related to differentiation. Levels of mRNA expression were normalized to internal control GAPDH, expressed as mean ± SEM. *P < 0.05 vs. Ad-GFP (n = 4); ^#^P < 0.05 vs. Ad-GFP with nesfatin-1 (n = 3–4). Each experiment was repeated at least 3 times. C: control; D: differentiation; N: nesfatin-1; L: leucine.

To investigate whether nesfain-1 promotes differentiation of primary brown adipocytes via mTOR signaling, we administrated leucine and nesfatin-1 alone or together before assaying brown adipocyte markers UCP1 and PGC1α. As shown in Fig. 6A, leucine blocked the nesfatin-1-induced up-regulation of both UCP1 (p = 0.003) and PGC1α (p = 0.002) in primary brown adipocytes. Levels of UCP1 and PGC1α mRNAs in brown adipocytes treated with both leucine and nesfatin-1 demonstrated no change relative to those in brown adipocytes treated with control vehicle.

Further, activation mTOR signaling pathway by deletion of *Tsc1* restrained the nesfatin-1-induced up-regulation in UCP1 (p = 0.024), PGC1α (p = 0.019), COX8B (p = 0.005), ATP5B (p = 0.005) in primary brown adipocytes (Fig. 6B). mRNA levels of these brown adipocyte marker genes demonstrated no difference compared with the brown adipocytes treated with control GFP adenovirus.

## Discussion

The present study demonstrates that nesfatin-1 is present in brown adipocytes and that nesfatin-1 promotes differentiation of primary brown adipocytes likely through the mTOR dependent mechanism. This conclusion is supported by the following observations: (1) NUCB2/nesfatin-1 mRNA and protein were detected in brown adipocytes. (2) Levels of nesfatin-1 decreased during the differentiation of primary brown adipocytes. (3) Nesfatin-1 up-regulated mRNA levels of brown adipocyte markers such as UCP1, UCP3, PGC1α, and PRDM16, while enhancing the expression of the lipolysis-related gene ATGL, and reducing expression of lipogenesis-related genes such as ACC, Fasn, and perilipin. (4) mTOR activity measured by S6 phosphorylation decreased during brown adipocyte differentiation. (5) Activation of mTOR attenuated the differentiation of primary brown adipocytes. (6)Treatment with leucine or deletion of *Tsc1* blocked brown adipocyte differentiation induced by nesfatin-1.

Brown adipose tissue regulates non-shivering thermogenesis using glucose and fatty acid as fuel to dissipate energy as heat driven by UCP1 located on the inner membrane of mitochondria. This feature renders brown adipose tissue a promising target for therapy of obesity and diabetes. Recent research has focused on regulation of the phenotypic switch between white and brown adipocytes. Induction of browning involves the transformation of white adipocytes into brown like adipocytes called brite cells or beige cells^11^. In addition to cold exposure and sympathetic mimetics such as the β3- adrenergic receptor agonists, factors that have been reported to promote thermogenesis in brown adipose tissue include thyroid hormone^12^, 5′-AMP-activated protein kinase(AMPK) activator^13^, fibroblast growth factor 21(FGF21)^14^, and bone morphogenetic protein 7(BMP7)^15^.

The current study identifies nesfatin-1 as an important molecule in the regulation of brown adipocyte differentiation and utilization of triglyceride. This finding extends the action of nesfatin-1 to brown adipocytes and provides further evidence supporting its critical role in the regulation of lipid homeostasis. Consistent with this observation, previous studies have demonstrated that nesfatin-1 inhibits lipid accumulation in 3T3-L1 cells^16^ and hepatocytes^17^, as well as reduces circulating fatty acid levels in diet-induced obese mice^17^,^18^. NUCB2/nesfatin-1 is expressed abundantly in the X/A like cells of gastric mucosa and may be released into the circulation to exert actions in both central and peripheral tissues. The presence of NUCB2/nesfatin-1 in brown adipocytes revealed by this study suggests an autocrine/paracrine function for this hormone. Potential autocrine/paracrine actions of NUCB2/nesfatin-1 are further supported by detection of this peptide in tissues such as brain, pancreas, gut, testis and white adipocytes. The relationship between the circulating nesfatin-1 secreted from the X/A like cells and brown adipocyte nesfatin-1 remains to be investigated. For example, future investigation should aim to examine whether circulating nesfatin-1 affects the production of this molecule in brown adipocytes. This examination may provide partial explanation for the discrepancy of our observations that nesfatin-1 activates thermogenesis while its mRNA and protein levels is down-regulated during brown adipocyte differentiation.

Conventional brown adipocytes share the same lineage with skeletal muscle expressing myogenic factor 5. Recent research demonstrates that white and brown adipocytes are able to transform reciprocally. Responding to cold exposure^19^,^20^,^21^, adrenergic stimulation^22^ or certain other stimuli, white adipocytes transform to brown-like adipocytes called brite cells or beige cells^11^. Interestingly, brite cells share several transcriptional factors with brown adipocytes, such as T-box15^23^. They also express several transcriptional features of white adipocytes, such as transcriptional factor 21 and homeoboxC8^24^,^25^. The main transcriptional pathway determining the differentiation of brown adipocytes or white adipocytes has been actively investigated^26^. Among these transcriptional factor, PR domain containing 16 (PRDM16) determines the transcriptional program of brown adipocytes rather than skeletal myocytes and white adipocytes^27^. The observation that nesfatin-1 significantly increases level of PRDM16 further confirms the effect of this hormone in the stimulation of brown adipocyte phenotype.

As a critical molecule integrating organism signals such as growth factors and nutrients with cellular growth, mechanistic target of rapamycin (mTOR) is critical for the differentiation of white and brown adipogenesis^13^,^28^. mTOR positively regulates commitment of mesenchymal stem cells to white adipogenic program^28^. In contrast, temporal control of mTOR is required for a proper brown adipocyte differentiation. Early activation of the mTOR signaling followed by a subsequent down-regulation of this signaling pathway seems crucial for a complete differentiation of brown phenotype. Inhibition of mTOR signaling by rapamycin and siRNA decreases cell proliferation and precludes brown adipocyte differentiation^13^. However, subsequent down-regulation of mTOR signaling by prolonged activation of AMPK is required for the fully differentiated brown adipocyte phenotype^13^. Consistently, our studies show an oscillatory mTOR signaling during brown adipocyte differentiation manifested by a temporal alteration in the phosphorylation of 4EBP1. By interacting with eIF4E, 4EBP1 inhibits complex assembly and represses protein translation. Activation of mTOR phosphorylates 4EBP-1, leading to the release of elF4E and subsequent initiation of protein translation^29^. 4EBP-1 knockout mice possess a significant increased amount of brown adipose tissue^30^. In 4EBP-1 knockout MEF cells, PGC1α expression increases significantly whereas exogenous 4EBP1 expression suppresses PGC1α levels^31^.

In our previous studies, activation of mTOR signaling by deletion of tuberous sclerosis complex 1 (Tsc1) decreases the quantity of brown adipocytes, while increasing cells with the morphological and genetic characteristics of white adipocytes in interscapular brown adipose tissue^32^. The current study suggests an inhibitory effect of nesfatin-1 on mTOR signaling manifested by decrement of phosphorylation of S6 and S6K1. Further, activation of mTOR signaling by either leucine or deletion of *Tsc1* significantly attenuates the effect of nesfatin-1 on brown adipocyte. Therefore, reduction in mTOR signaling may contribute to the stimulatory action of nesfatin-1 on brown adipocyte differentiation. Consistent with this observation, nesfatin-1 has been demonstrated to inhibit mTOR signaling in the dorsal motor nucleus of the vagus^33^ and to activate AMPK signaling in hepatocytes^17^. Activation of AMPK is well recognized to inhibit mTOR signaling in a variety of cell type^34^. Taken together, these studies from ours and others suggest that mTOR may function as a critical intracellular molecule in the determination of brown adipocytes in response to either nutrients or hormones such as nesfatin-1.

Fatty acid is the main fuel utilized by brown adipocytes for thermogenesis. The balance between triglyceride and fatty acids is tightly regulated by a series of enzymes critical for lipogenesis and lipolysis. Our study suggests that nesfatin-1 promotes lipid utilization by up-regulating ATGL, while inhibiting lipogenesis by down-regulating ACC and Fasn. ATGL is essential in generating fatty acids for oxidation and UCP-1 activation for thermogenesis. Global deletion of ATGL in mice down-regulates the insulin induced glucose clearance in brown adipocyte while increases its lipid content^35^. Adipocyte-specific ablation of ATGL converts brown adipocytes to white-like adipocytes. All these observation indicate that ATGL may involve in maintaining the phenotype of brown adipocytes^36^. Perilipin locates on the surface of lipid droplets, and is essential for dynamic alternation of lipid droplets^37^. Basal perilipin stabilizes lipid droplets, preventing lipolysis, while phosphorylated perilipin activated by PKA facilities lipolysis^38^. Perilipin knockout induced an increase in fatty acid release rate in brown adipocytes and white adipocytes^39^,^40^.The current observation of decrement in perilipin expression indicates an increase in lipid droplet utilization induced by nesfatin-1. Adipose tissue specific ablation of Fasn increases energy expenditure and browning of subcutaneous white adipose tissue^41^. Our studies thus reveal that nesfatin-1 contributes to lipid homeostasis by controlling the fine tuning between utilization of fatty acid and lipogenesis in brown adipocytes.

In summary, this study demonstrates that nesfaitn-1 promotes brown adipocyte differentiation likely through mTOR signaling pathway. This finding suggests that nesfatin-1 may be a promising strategy for treatment of obesity.

## Materials and Methods

### Materials

Phospho-S6 (ser235/236) and S6 rabbit monoclonal antibodies, UCP1 rabbit monoclonal antibody, and phoshpo-S6k1 mouse monoclonal antibody were purchased from Cell Signaling Technology (Beverly, MA). Mouse anti-GAPDH was purchased from Santa Cruz Biotechnology (Santa Cruz, CA). Nesfatin-1 and rabbit anti- nesfatin-1/NUCB2 polyclonal antibody were purchased from Phoenix Pharmaceuticals (Burlingame, CA). Insulin, indomethacin, isobutylmethylxanthine, dexamethasone, leucine, and collagenase I were from Sigma Aldrich (St. Louis, MO). IRDye-conjugated affinity purified anti-rabbit and anti-mouse IgGs were purchased from Rockland (Gilbertsville, PA). Trizol reagent and the reverse transcription (RT) system were purchased from Invitrogen Inc. (Grand Island, NY).

### Animals and Animal Care

*Tsc1*^lox/lox^ mice in which exons 17 and 18 of *Tsc1* gene are flanked by loxP sites by homologous recombination, were purchased from The Jackson Laboratory (Bar Harbor, ME). Normal and Tsc1^lox/lox^ C57BL/6J mice were housed on a 12:12-h light/dark cycle. Regular chow and water were available *ad libitum*. The animals used in this study were handled in accordance with the Guide for the Care and Use of Laboratory Animals published by the National Institutes of Health (publication no. 85-23, revised 1996). All experimental protocols were approved by the Animal Care and Use Committee of Peking University Health Science Center.

### Culture of Primary Brown Adipocytes

Brown adipose tissues were harvested from interscapular brown adipose tissue of neonatal C57BL/6J mice and brown pre-adipocytes were isolated by digesting the tissue with collagenase and mechanistic dispersion as described previously^11^. Isolated cells were randomly plated in tissue-culture dishes in DMEM supplemented with 20% FBS. After 4 h of culture at 37 °C, cells were rinsed twice with PBS, after which 70% of the initial cells were attached to the dish, forming a monolayer. Isolated brown pre-adipocytes were cultured in DMEM supplemented with 20% FBS for proliferation. To induce brown adipocyte differentiation, cells were cultured for 2 days in 10% FBS-DMEM supplemented with 20 nmol/L insulin, 1 nmol/L T3, 12.5 mmol/L indomethacin, 0.5 mmol/L isobutylmethylxanthine, and 2 mg/mL dexamethasone. On days 3–8, the induction medium was substituted by maintenance medium consisting of DMEM supplemented with 20 nmol/L insulin and 1 nmol/L T3. For the undifferentiated control, brown pre-adipocytes were cultured in 10% FBS-DMEM without other supplements. During differentiation, nesfatin-1 and/or leucine were added to the differentiation medium once a day, while saline was added to the control group.

### Adenovirus Infection

The Cre adenoviruses were expanded, titrated in 293 cells, and purified by cesium chloride methods as described previously^42^. For adenovirus-mediated gene transfer, brown adipocytes were infected with 10^6 titer adenovirus for 48 h. Infection efficiency was judged by green fluorescent protein (GFP) expression observed under the microscope. Infected primary brown pre-adipocytes were differentiated for 4 days and then harvested for subsequent analysis.

### RNA Extraction and Quantitative Real-Time PCR Analysis

Total RNA was isolated using the TRIzol reagent. Reverse transcription was performed using the RT system according to the manufacturer’s instruction. PCR was conducted in a 25 μL volume containing 2.5 μL cDNA, 5 mM MgCl2, 0.2 mM dNTPs, 0.2 μM each primer, 1.25 U AmpliTaq Polymerase and 1 μL 800 × diluted SYBRGreen I stock using the Mx3000 multiple quantitative PCR system (Strata gene, La Jolla, CA). PCR reactions were performed in duplicate, and each experiment was repeated three to five times.

mRNA expression was quantified using the comparative cross threshold (CT) method. The CT value of the housekeeping gene GAPDH was subtracted from the CT value of the target gene to obtain ΔCT. The normalized fold changes of target gene mRNA expression were expressed as 2^−ΔΔCT^, where ΔΔCT equals to ΔCT sample-ΔCT control.

### Western Blot Analysis

Cultured cells were harvested and homogenized in ice-cold fractionation buffer. The cell lysate was treated with ultrasound for 3 seconds three times, then centrifuged at 12000*rpm* for 10 min at 4 °C. After centrifugation, the supernatant was used for Western blot analysis. Protein concentration was measured by Bradford’s method. A total of 80 μg protein from each sample was loaded onto SDS-PAGE gels. Proteins were transferred to polyvinylidene fluoride membranes. The membranes were incubated for 1 h at room temperature with 5% fat-free milk in Tris buffered saline containing Tween-20, followed by incubation overnight at 4 °C with the individual primary antibody. Specific reaction was detected using IRDye conjugated second antibody and visualized using the Odyssey infrared imaging system (LI-COR Biosciences, Lincoln, NE). Quantification of image density in pixel was performed using the Odyssey infrared imaging system (LI-COR Biosciences, Lincoln, NE).

### Statistical Analysis

Data were expressed as mean ± SEM. Data analysis used GraphPad Prism software. One-way ANOVA, Student-Newman-Keul test (comparisons between multiple groups), or unpaired Student t test (between two groups) were used as appropriate. P < 0.05 denotes statistical significance.

## Additional Information

**How to cite this article**: Wang, Y. *et al*. Nesfatin-1 promotes brown adipocyte phenotype. *Sci. Rep.* **6**, 34747; doi: 10.1038/srep34747 (2016).

**Publisher’s note**: Springer Nature remains neutral with regard to jurisdictional claims in published maps and institutional affiliations.
